# Constitutive Expression of *OsIAA9* Affects Starch Granules Accumulation and Root Gravitropic Response in *Arabidopsis*

**DOI:** 10.3389/fpls.2015.01156

**Published:** 2015-12-22

**Authors:** Sha Luo, Qianqian Li, Shanda Liu, Nicholaas M. Pinas, Hainan Tian, Shucai Wang

**Affiliations:** Key Laboratory of Molecular Epigenetics of MOE, Northeast Normal UniversityChangchun, China

**Keywords:** OsIAA9, auxin signaling, gravitropism, lateral root formation, *Arabidopsis thaliana*, *Oryza sativa*

## Abstract

*Auxin*/*Indole-3-Acetic Acid* (*Aux*/*IAA*) genes are early auxin response genes ecoding short-lived transcriptional repressors, which regulate auxin signaling in plants by interplay with Auxin Response Factors (ARFs). Most of the Aux/IAA proteins contain four different domains, namely Domain I, Domain II, Domain III, and Domain IV. So far all *Aux*/*IAA* mutants with auxin-related phenotypes identified in both *Arabidopsis* and rice (*Oryza sativa*) are dominant gain-of-function mutants with mutations in Domain II of the corresponding Aux/IAA proteins, suggest that Aux/IAA proteins in both *Arabidopsis* and rice are largely functional redundantly, and they may have conserved functions. We report here the functional characterization of a rice *Aux*/*IAA* gene, *OsIAA9*. RT-PCR results showed that expression of *OsIAA9* was induced by exogenously applied auxin, suggesting that *OsIAA9* is an auxin response gene. Bioinformatic analysis showed that OsIAA9 has a repressor motif in Domain I, a degron in Domain II, and the conserved amino acid signatures for protein-protein interactions in Domain III and Domain IV. By generating transgenic plants expressing *GFP-OsIAA9* and examining florescence in the transgenic plants, we found that OsIAA9 is localized in the nucleus. When transfected into protoplasts isolated from rosette leaves of *Arabidopsis*, OsIAA9 repressed reporter gene expression, and the repression was partially released by exogenously IAA. These results suggest that OsIAA9 is a canonical Aux/IAA protein. Protoplast transfection assays showed that OsIAA9 interacted ARF5, but not ARF6, 7, 8 and 19. Transgenic *Arabidopsis* plants expressing *OsIAA9* have increased number of lateral roots, and reduced gravitropic response. Further analysis showed that *OsIAA9* transgenic *Arabidopsis* plants accumulated fewer granules in their root tips and the distribution of granules was also affected. Taken together, our study showed that OsIAA9 is a transcriptional repressor, and it regulates gravitropic response when expressed in *Arabidopsis* by regulating granules accumulation and distribution in root tips.

## Introduction

In *Arabidopsis*, *Aux/IAA* genes are one of the early auxin response gene families (Hagen and Guilfoyle, 2002). Aux/IAA proteins are short-lived transcription repressors that involve in the regulation of auxin signaling (Guilfoyle and Hagen, 2007; Guilfoyle, 2015). Most of the Aux/IAA proteins contain four conserved domains, namely, Domain I, Domain II, Domain III, and Domain IV. Domain I is an active repression domain containing a conserved LxLxL motif. Domain II contains a conserved degron and is responsible for the stability of Aux/IAA proteins. Domains III and IV are similar to the conserved C-terminal dimerization domain of Auxin Response Factors (ARFs), and are required for homo dimerization among Aux/IAA proteins, and hetero dimerization between Aux/IAA proteins and ARFs (Ulmasov et al., 1997, 1999; Ramos et al., 2001; Tiwari et al., 2001, 2003, 2004; Dreher et al., 2006; Nanao et al., 2014).

Aux/IAA proteins regulate auxin signaling by interplay with ARFs (Guilfoyle and Hagen, 2007; Guilfoyle, 2015). When cellular auxin levels are low, Aux/IAA proteins are stable, and they can form dimers with ARF activators that bound on the TGTCTC Auxin Response Elements (AuxREs) in the promoter regions of the auxin response genes, thus inhibiting the expression of auxin response genes. Elevated cellular auxin levels will result in the activation of auxin receptor TIR1 (Dharmasiri et al., 2005; Kepinski and Leyser, 2005), leading to the ubiquitylation and then degradation of Aux/IAA proteins via the 26S proteasome (Tan et al., 2007), thus allowing the ARF activators to activate auxin response genes (Ulmasov et al., 1999; Tiwari et al., 2001, 2004; Guilfoyle and Hagen, 2007).

So far all the *aux*/*iaa* mutants identified in *Arabidopsis*, including single T-DNA insertion mutants, double, and triple mutants of closely related *Aux*/*IAA* genes, showed no visible developmental defects (Overvoorde et al., 2005), suggesting that *Aux/IAA* genes are largely redundant functionally. On the other hand, several different types of phenotypes were observed in the dominant *aux*/*iaa* mutants. for example, the *iaa18-1* mutant has aberrant cotyledon placement in embryos (Ploense et al., 2009), the *axr2-1*/*iaa7* mutant has agravitropic root and shoot growth, and a short hypocotyl and stem (Nagpal et al., 2000), the *iaa16-1* mutant has restricted adult plant growth and abolished fertility in homozygous (Rinaldi et al., 2012), the *iaa18* and *iaa28* mutants are severely defective in lateral root formation (Rogg et al., 2001; Uehara et al., 2008), and the *slr-1*/*iaa14* mutant completely lacks lateral roots (Fukaki et al., 2002). Some of the dominant gain-of-function *iaa* mutants even have opposite phenotypes. For example, the *axr3*/*iaa17* mutant has defects in root hair development, while *shy2*/*iaa3* mutant has longer root hairs (Knox et al., 2003). The *shy2*/*iaa3* mutant also has other phenotypes including enlarged cotyledons, short hypocotyls, and altered auxin-regulated root development (Tian and Reed, 1999; Tian et al., 2002). These results suggest that stability of Aux/IAA proteins is crucial for their functions in regulating plant growth and development.

Consistent with the fact that Aux/IAA protein are unstable proteins, *Arabidopsis* transgenic plants expressing wild type *Aux*/*IAA* genes from *Arabidopsis* and grape (*Vitis vinifera*) are also morphological indistinguishable from wild type plants (Park et al., 2002; Fujita et al., 2012; Kohno et al., 2012). However, auxin-related phenotypes were observed in *Arabidopsis* transgenic plants expressing mutated *Aux*/*IAA* gene with a mutation in Domain II, or *Aux*/*IAA* gene lacking Domain II (Park et al., 2002; Fukaki et al., 2005; Sato and Yamamoto, 2008).

Phenotypic changes were observed in knock-down mutants of *Aux*/*IAA* genes in tomato (*Solanum lycopersicum*) (Wang et al., 2005a; Bassa et al., 2012; Deng et al., 2012; Su et al., 2014), and expressing wild type *PtrIAA14.1*, a poplar *Aux*/*IAA* gene in *Arabidopsis* resulted in phenotypic changes (Liu et al., 2015), suggesting the functions of *Aux*/*IAA* from at least some plant species may different from that in *Arabidopsis*.

In rice (*Oryza sativa*), however, all the identified *Aux*/*IAA* mutants with auxin-related phenotypes are dominant gain-of-function mutants with mutations in the Domain II of corresponding Aux/IAA proteins (Jun et al., 2011; Zhu et al., 2011; Kitomi et al., 2012), and auxin-related phenotypes were also observed in transgenic rice plants expressing mutated or dominant mutation-type rice *Aux*/*IAA* genes (Nakamura et al., 2006; Song and Xu, 2013), suggesting that Aux/IAA proteins in rice may regulate auxin signaling in a way similar to those in *Arabidopsis*.

We report here the characterization of *OsIAA9*, a rice Aux/IAA gene. Expression of *OsIAA9* was greatly induced by exogenously supplied IAA. Bioinformatics and protoplast transfection assay results showed that OsIAA9 is a canonical Aux/IAA protein. When expressed in *Arabidopsis* in a wild type form, however, *OsIAA9* affected lateral root formation and root gravitropic response.

## Materials and Methods

### Plant Materials and Growth Conditions

The Japonica rice (*Oryza sativa*) variety *Nipponbare* was used for Auxin treatment and *OsIAA9* gene cloning. The *Arabidopsis thaliana* (*Arabidopsis*) ecotype Columbia-0 (Col-0) was used for plant transformation and protoplasts isolation. *DR5:GUS* transgenic plants were in Col background (Wang et al., 2005b).

For auxin treatment and RNA isolation from rice seedlings, rice seeds were germinated and grown on water for 10 d, and treated with 10 μM IAA for 4 h before RNA was isolated. For plant transformation and protoplasts isolation, *Arabidopsis* seeds were sown directly into soil pots and kept in a growth room. For phenotypic analysis, *Arabidopsis* seeds were sterilized and sown on 0.8% (w/v) phytoagar solidified ½ MS (Murashige & Skoog) (Murashige and Skoog, 1962) plates with vitamins (PlantMedia) and 1% (w/v) sucrose, unless indicated otherwise. The plates were kept at 4°C in darkness for 2 days, and then moved into a growth room. Rice plants was grown at 28°C, and *Arabidopsis* plants at 20°C, with a 16 h/8 h (light/darkness) photoperiod.

### Phylogenetic Analysis

Closely related *Arabidopsis* and rice Aux/IAA proteins to OsIAA9 were identified by BLAST searching *Arabidopsis* and rice proteome database^1^ using the entire amino acid sequence of OsIAA9. Full-length amino acid sequences of OsIAA9 and closely related *Arabidopsis* and rice Aux/IAA proteins were subjected to phylogenetic analysis using “One Click” mode with default settings on Phylogeny^2^.

### Auxin Treatment

To examine the expression of *OsIAA9* in response to auxin, 10-day-old rice seedlings were treated with 10 μM IAA for 4 h in darkness on a shaker at 40 rpm. Samples were frozen in liquid N_2_ and kept at -80°C for RNA isolation.

### RNA Isolation and RT-PCR

Total RNA from rice and *Arabidopsis* seedlings was isolated by using the procedures described previously (Wang et al., 2014; Guo et al., 2015; Liu et al., 2015). cDNA was synthesized by Oligo(dT)-primed reverse transcription using the EazyScript First-Strand DNA Synthesis Super Mix (TransGen Biotech) according to the manufacturer’s instructions.

RT-PCR was used to examine the expression of *OsIAA9*, and *Arabidopsis* gene *ACTIN2* (*ACT2*) or rice gene *OsACT2* were used as controls for RT-PCR.

### Constructs

The reporters constructs *LexA-Gal4:GUS* and *Gal4:GUS*, and the effector constructs *GD*, *LD-VP*, *CAT*, and *ARFs* used for protoplast transfection were as described previously (Tiwari et al., 2003; Wang et al., 2005b, 2007; Liu et al., 2015).

To generate HA or GD tagged *OsIAA9* constructs for protoplast transfection assays, the full-length open-reading frame (ORF) of *OsIAA9* was amplified by RT-PCR using RNA isolated from rice seedlings, and cloned in frame with an N-terminal HA or GD tag into the *pUC19* vector under the control of the *35S* promoter (Tiwari et al., 2003; Wang et al., 2005b). The *35S:OsIAA9* construct in *pUC19* was digested with proper enzymes, and subcloned into the binary vector *pPZP211* (Hajdukiewicz et al., 1994) for plant transformation.

To generate GD tagged OsIAA9CTD for protoplast transfection, ORF sequence of OsIAA9CTD (corresponding to the amino acid residues 84–182) was amplified by PCR using *35S:OsIAA9* plasmids as template, and cloned in frame with an N-termianl GD tag into the *pUC19* vector under the control of the *35S* promoter.

To generate GFP tagged OsIAA9 for subcellular localization analysis of OsIAA9, GD tag in *35S:GD-OsIAA9* construct was replaced with a GFP tag, subcloned into the binary vector *pPZP211*, and used for plant transformation.

The primers used for gene cloning and gene expression analysis of *OsIAA9* are *OsIAA9-F*, 5′-CAACATATGGAGCTGGAGCTTGGGCT-3′, *OsIAA9-R*, 5′- CAACTTAAGTTAACCCAGTATCTTCAGGC -3′, and *OsIAA9CTD-F*, 5′-CAACATATGTCGGCGCGGCGGGCGT-3′. The primers used to amplify *ACT2* and *OsACT2* were described previously (Guo et al., 2015).

### Plant Transformation and Transgenic Plants Selection

About 5-week-old *Arabidopsis* plants with several mature flowers on the main inflorescence were transformed using the floral dip method (Clough and Bent, 1998). T1 seeds were sterilized and grown on 1/2 MS plates containing 50 μg/ml kanamycin to select transgenic plants. At least five transgenic lines with similar phenotypes were obtained. Phenotypes of transgenic plants were examined in the T1 generation, and confirmed in following several generations. Represent homozygous T3 or T4 transgenic plants were used for further analysis.

### Plasmid DNA Isolation, Protoplasts Isolation, Transfection, and GUS Activity Assays

The procedures for plasmids preparation, protoplast isolation, transfection, and GUS activity assay have been described previously (Wang et al., 2005b, 2007, 2008, 2014, 2015; Zhou et al., 2014; Guo et al., 2015; Liu et al., 2015). Briefly, plasmids of the reporter and effector genes were prepared using the GoldHi EndoFree Plasmid Maxi Kit (Kangwei), and co-trasfected into protoplasts isolated from rosette leaves of ∼4-week-old Col wild type *Arabidopsis* plants. The transfected protoplasts were incubated under darkness at room temperature for 20–22 h before GUS activities were measured using a SynergyTM HT microplate reader (BioTEK).

### Gravitropic Response Assays

Gravitropic response was measured as described by Li et al. (2011). Briefly, sterilized seeds were grown vertically on 1/5 MS plates for 4 days. The plates were photographed and the plates were then turned 90^0^. The plates were photographed again 1 day later. Angles of the angle between gravity and the root were measured using NIH Image J^3^.

### Starch Granule Staining

Starch granule was stained by following the procedures described by Li et al. (2011).

### Microscopy

Photographs of *Arabidopsis* seedlings were taken under a Motic K dissection microscope equipped with an EOS 1100D camera. Root length was then measured using NIH Image J. GFP florescence of *OsIAA9-GFP* transgenic *Arabidopsis* seedlings was examined under an Olympus FV1000 confocal microscope.

## Results

### *OsIAA9* is an Auxin Response Gene That Encodes a Canonical Aux/IAA Protein

Available experimental evidences suggest that functional mechanism of Aux/IAA may be conserved in rice and *Arabidopsis*. To test if this is the case, we decided to identify a canonical rice *Aux*/*IAA* gene, and study its functions in the regulation of auxin signaling and plant growth and development in *Arabidopsis*.

**FIGURE 1:**
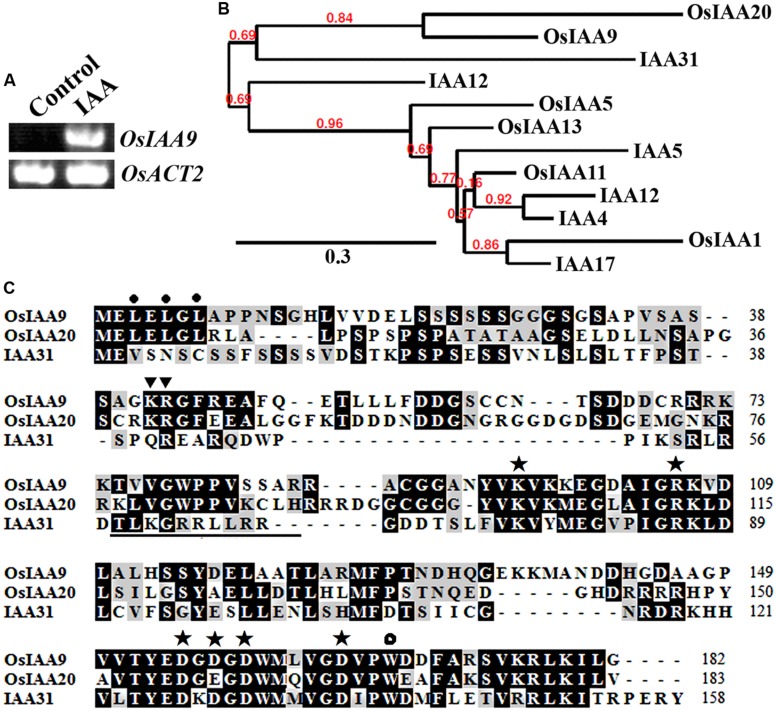
**OsIAA9 is a canonical Aux/IAA protein.** **(A)** Expression of *OsIAA9* in rice seedlings in response to IAA treatment. RNA was isolated from IAA treated rice seedlings and RT-PCR was used to examine the expression of *OsIAA9*. Expression of *OsACT2* was used as a control. **(B)** Phylogenetic analysis of OsIAA9 and some *Arabidopsis* and rice Aux/IAA proteins that have relative higher amino acid similarities with OsIAA9. The entire amino acid sequences were used to generate phylogenetic tree on Phylogeny (http://www.phylogeny.fr) by using the “One Click” mode with default settings. Branch support values are indicated above the branches. **(C)** Amino acid sequence alignment of OsIAA9, OsIAA20 and IAA31. Domain II is underlined. The KR residues between Domain I and II that are crucial for protein degradation are indicated by triangles, the L residues in the LxLxL repression motif are indicated by closed circles, the conserved residues in Domain III and IV that are crucial for protein–protein interaction are indicated by asterisks, and the conserved W residue in OsIAA23 that is crucial for protein–protein interaction is indicated by open circle.

*OsIAA9* was chosen because it is one of the Aux/IAA genes whose expression was highly induced by 2,4-D, a synthesized auxin (Jain et al., 2006). As shown in **Figure 1A**, expression of *OsIAA9* was also highly induced by IAA, a natural occurred auxin. By using the entire amino acid sequence of OsIAA9 to BLAST rice and *Arabidopsis* protein database^4^, we identified and selected several Aux/IAA proteins that have relative higher amino acid sequence similarity to OsIAA9. Phylogenetic analysis showed that OsIAA9 and OsIAA20 are paralogs. The most closely related *Arabidopsis* Aux/IAA to OsIAA9 is IAA31 (**Figure 1B**), an Aux/IAA protein with mutated Domain II (**Figure 1C**). These results are largely consistent with that reported by Jain et al. (2006).

Previously experiment showed that canonical Aux/IAA proteins contain an LxLxL repressor motif in Domain I, a GWPPV degron core sequence in Domain II, conserved KR residues between Domain I and Domain II that are crucial for the degradation of *Arabidopsis* Aux/IAA proteins, some conserved amino acid residues in Domain III and Domain IV that are require for protein–protein interaction among Aux/IAA proteins or between Aux/IAA proteins and ARFs (Tiwari et al., 2001, 2003, 2004; Dreher et al., 2006; Nanao et al., 2014). As shown in **Figure 1C**, OsIAA9 has all the features for a canonical Aux/IAA protein. In addition, OsIAA9 also has the conserved W residue found in OsIAAs and OsARFs that is crucial for protein-protein interaction (Ni et al., 2014).

### OsIAA9 is a Transcription Repressor and its Stability is Affected by Auxin

**FIGURE 2:**
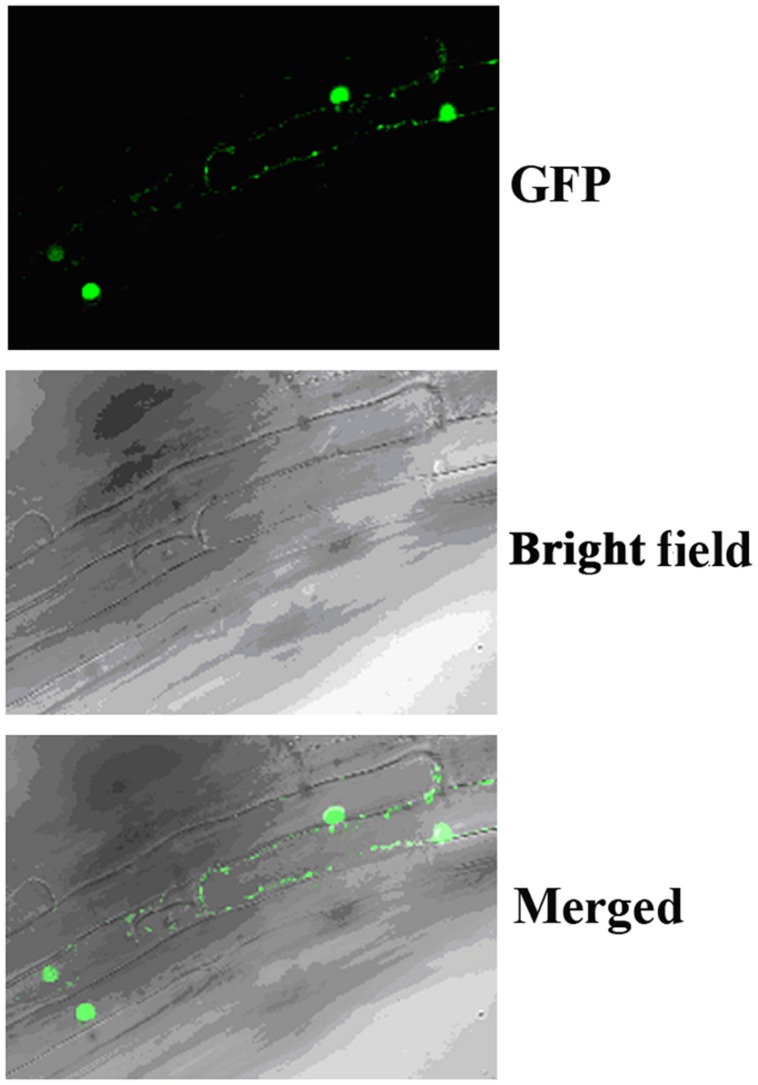
**Subcellular localization of OsIAA9.** Root tips of transgenic *Arabidopsis* seedlings expression *GFP-OsIAA9* under the control of the *35S* promoter were examined under a florescence microscope. **(Upper)** GFP channel, **(middle)** bright field image, **(lower)** merged.

In *Arabidopsis*, canonical Aux/IAA proteins are short-lived proteins whose stability is affected by auxin, and they function as transcription repressors. To examine if OsIAA9 is a transcription repressor and its stability is affected by auxin, we first examine it subcellular localization by examining transgenic *Arabidopsis* plants expressing *GFP-OsIAA9* under the control of the *35S* promoter. As shown in **Figure 2**, OsIAA9 is predominantly localized in nucleus.

**FIGURE 3:**
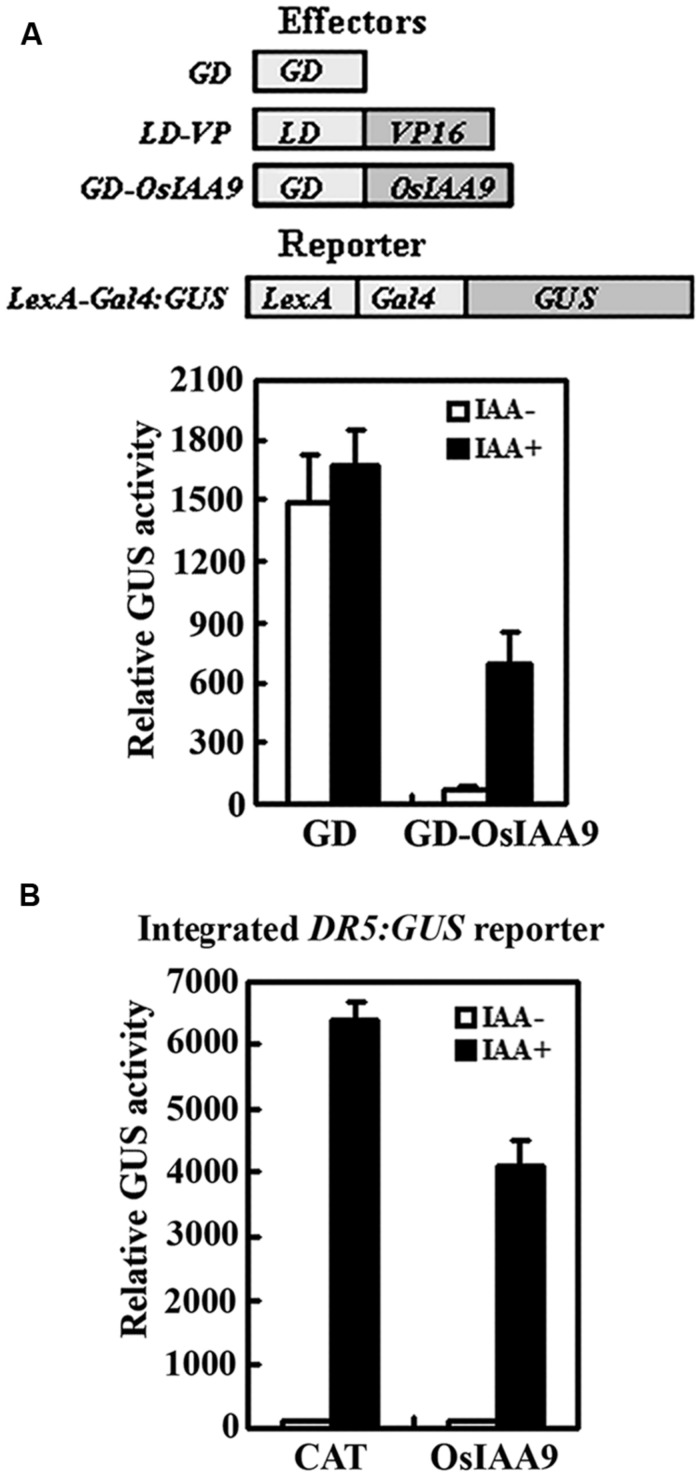
**OsIAA9 is a transcriptional repressor and its stability is affected by auxin.** Repression of *LexA-Gal4:GUS* reporter gene **(A)**, and the integrated *DR5:GUS* reporter gene **(B)** by OsIAA9. Effectors and reporters (drawn on the top of **A**) or effectors plasmids alone were transfected into protoplasts isolated from Col wild type, or *DR5:GUS* transgenic plants, and incubated in the presence and absence of 1 μM IAA for 20–22 h, then GUS activity was assayed. The *35S:CAT* (chloramphenicol acetyltransferase) plasmids were used as a control. Data represent the mean ± SD of three replicates.

We then examined if OsIAA9 functions as transcriptional repressor by using protoplast transfection assays. Plasmids of effector gene *GD-OsIAA9* or control gene *GD*, activator gene *LD-VP*, and the reporter gene *LexA-GAL4:GUS* were contransfected into protoplasts, and GUS activities were measured after the transfected protoplasts were incubated in the presence and absent of 1.0 μM IAA. As shown in **Figure 3A**, in the absence of IAA, co-transfection of control gene *GD* and activator gene *LD-VP* activated the reporter gene, while co-transfection of effector gene *GD-OsIAA9* and activator gene *LD-VP* resulted in repression of the reporter gene. In the presence of IAA, the repression on the expression of the reporter gene by co-transfected *OsIAA9* gene was partially released (**Figure 3A**), indicating that OsIAA9 is a transcription repressor, and it is unstable in the presence of auxin. When transfected into protoplasts with an integrated auxin response reporter gene *DR5:GUS*, OsIAA9 repressed the expression of the reporter gene (**Figure 3B**), suggesting that OsIAA9 regulates auxin response gene expression.

### OsIAA9 Interacts With ARF5 in Plant Cells

In *Arabidopsis*, Aux/IAA proteins regulate auxin signaling through interacting with ARF activators. So far only five ARF activators in *Arabidopsis* have been shown to be transcriptional activators (Wang et al., 2005b). Having shown that OsIAA9 is a transcriptional repressor and it regulates auxin reporter gene expression (**Figure 3**), we wanted to further examine if OsIAA9 interacts with any of the ARF activators by using protoplast transfection assays.

**FIGURE 4:**
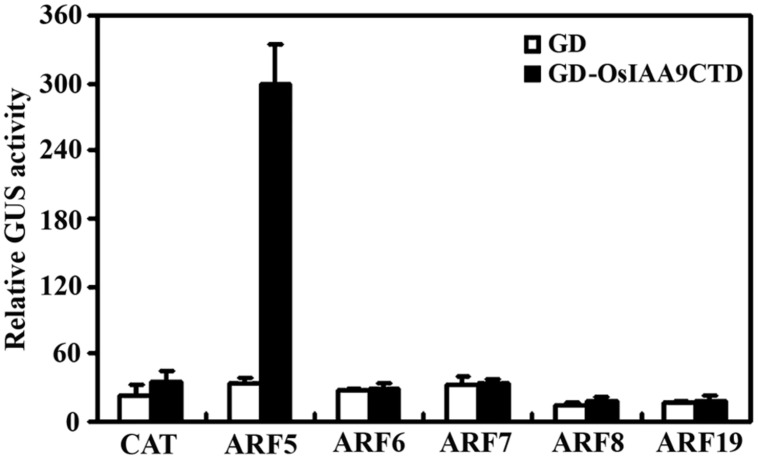
**OsIAA9 interacts with ARF5 in plant cells.** Plasmids of effectors *GD-OsIAA9CTD* and *ARFs*, and reporter *Gal4:GUS* were co-transfected into protoplasts and incubated for 20–22 h, then GUS activity was assayed. Data represent the mean ± SD of three replicates.

Because Aux/IAA proteins have been shown to be active transcription repressor, and Domain III and IV are the domains that required for the interaction among Aux/IAA proteins and between Aux/IAA protein and ARFs, we made a *GD-OsIAA9CTD* construct by fusing OsIAA9 C-terminal Domain (Domain III and IV) with GD, and cotransfected it with ARF activator effector genes and the reporter gene *Gal4:GUS* into protoplasts. *GD* gene was cotransfected as a control. GUS activity was measured after incubation. As shown in **Figure 4**, cotransfection of *GD* gene with all the ARF activator genes does not have any effects on the expression of the reporter gene, while cotransfection of *GD-OsIAA9CTD* gene with *ARF5* gene, but not other ARF activator genes activated *Gal4:GUS* reporter, suggesting that OsIAA9 specifically interacts with ARF5.

### Expression of *OsIAA9* in *Arabidopsis* Affects Lateral Root Formation and Root Gravitropic Response

**FIGURE 5:**
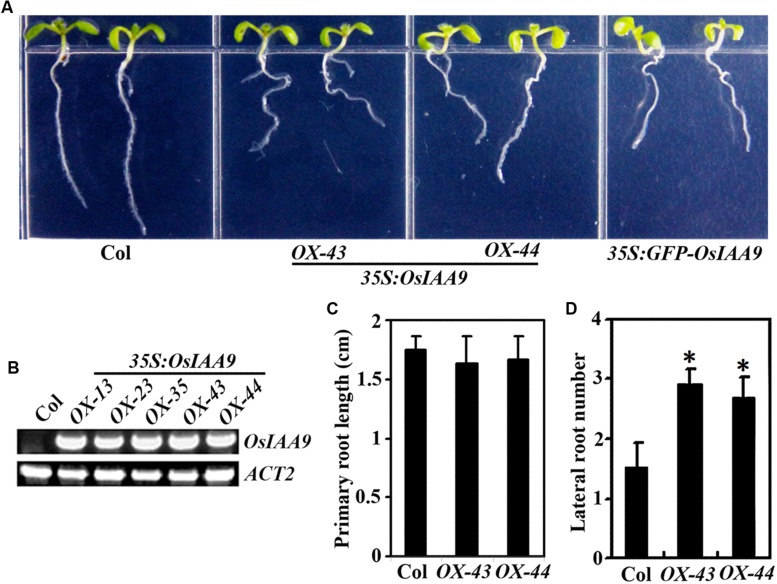
**Morphologies of *Arabidopsis* transgenic plant seedlings expressing *OsIAA9*.** **(A)** Seven-day-old Col wild type and transgenic plant seedlings. **(B)** Expression of *OsIAA9* in transgenic plants. RNA was isolated from *OsIAA9* transgenic *Arabidopsis* seedlings and RT-PCR was used to examine the expression of *OsIAA9*. Expression of *ACT2* was used as a control. **(C)** Primary root length of 7-day-old wild type and transgenic plants. **(D)** Lateral root number of 10-day-old wild type and transgenic plants. Data in **(C)** and **(D)** represent the mean ± SD of 12–15 seedlings. ^∗^, significantly different from Col wild type seedlings (*p* < 0.0001).

The results described above indicate that OsIAA9 is a canonical Aux/IAA protein. *Arabidopsis* transgenic plants overexpressing wild type *Arabidopsis* and grape Aux/IAA genes are morphologically similar to wild type plants (Park et al., 2002; Fujita et al., 2012; Kohno et al., 2012). However, expression of *PtrIAA14.1*, a canonical Aux/IAA protein encoding wild type poplar Aux/IAA gene in *Arabidopsis* resulted in auxin-related phenotypes including semi-draft with increased number of branches, down-curling leaves, and greatly reduced fertility (Liu et al., 2015). To further explore if OsIAA9 functions similar to canonical Aux/IAA proteins in *Arabidopsis*, we generated transgenic plant expressing *OsIAA9* under the control of the *35S* promoter (*35S:OsIAA9*). As shown in **Figure 5A**, transgenic *Arabidopsis* plants have reduced root gravitropic response as indicated by the root orientations. Expression of *OsIAA9* in the transgenic plants was confirmed by RT-PCR (**Figure 5B**). Quantitative analysis showed that root elongation is largely unaffected in the *Arabidopsis* transgenic plants expressing *OsIAA9* (**Figure 5C**). However, the transgenic plants produced more lateral roots (**Figure 5D**).

Transgenic plants expressing *GFP-OsIAA9* also resulted in abnormal root gravitropic response (**Figure 5A**), indicating that GFP-OsIAA9 protein is functional, thus the plants were used to examine subcellular localization of OsIAA9 (**Figure 2**).

**FIGURE 6:**
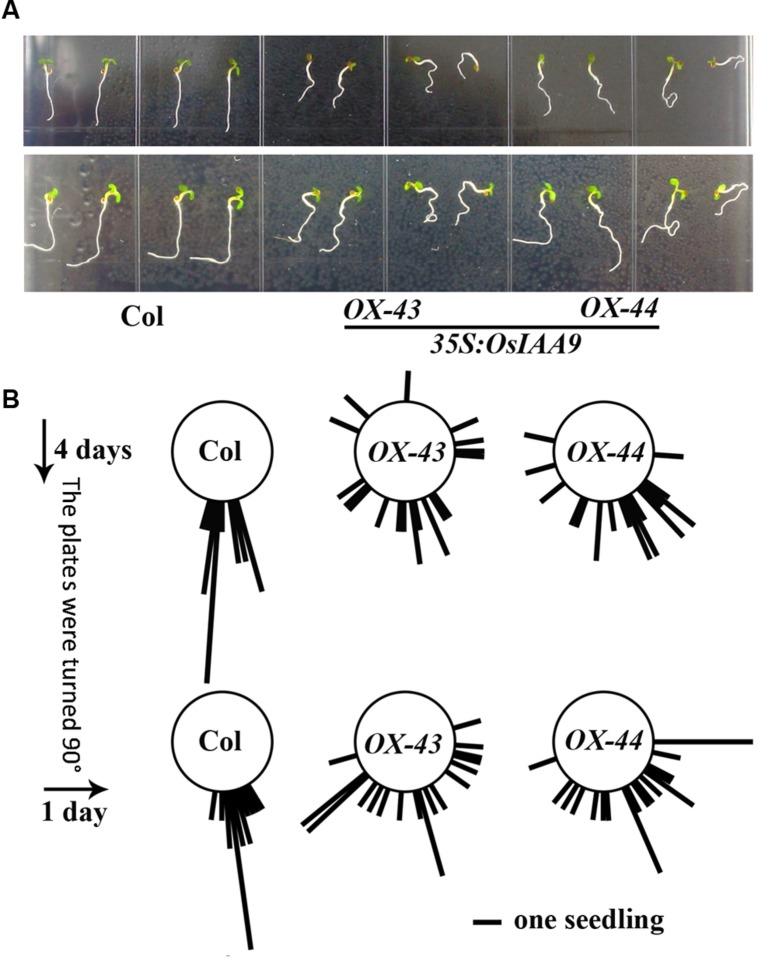
**Expression of *OsIAA9* in *Arabidopsis* affects root gravitropic response.** **(A)** Response of Col wild type (left) and *OsIAA9* transgenic seedlings (middle and right) to a directional change of gravity. Up panel: before the plates were turned, low panel: 1 day after the plates were turned. Seedlings were grown on vertically plates for 4 days, and then the plates were turned 90^0^ and kept for 1 days. **(B)** Quantification of response of Col wild type and OsIAA9 transgenic plant seedlings to a directional change of gravity. The angle between the gravity vector and the root were measured when the seedlings were grown vertically for 4 days and 1 day after the plates were turn 90^0^. Bar represents one seedling.

To further examine the roles of OsIAA9 in regulating root gravitropic response, we tested the gravitropic response of Col wild type and *OsIAA9* transgenic *Arabidopsis* seedlings to changed gravity. Seedlings were grown on vertical plates for 4 days, and pictured to measure the root directions. The plates were then turned 90^0^ to change the gravity, photographs were taken again after 24 h. As shown in **Figure 6A**, wild type seedlings grew straight down toward the gravity direction before and after the plates were turned, whereas roots of transgenic seedling were in random directions before and after the plates were turned. Quantitative results of the root direction further confirmed our observation (**Figure 6B**).

### Starch Granules Accumulation is Affected in the *OsIAA9* Transgenic *Arabidopsis* Seedlings

**FIGURE 7:**
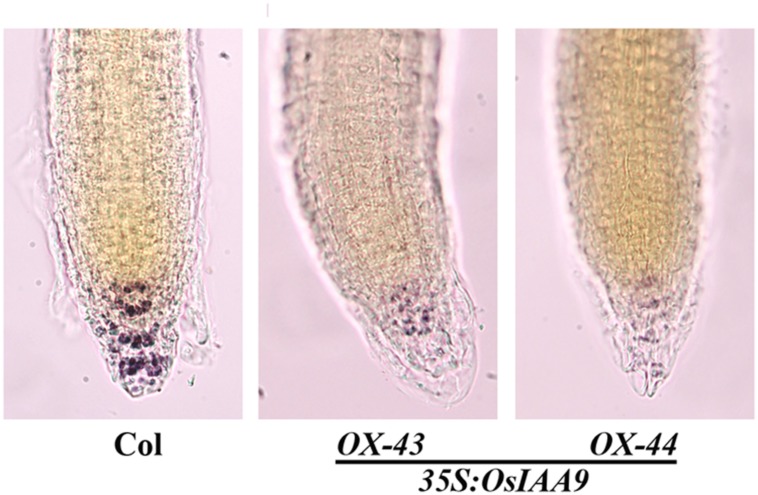
**Starch Granules formation in root tips of *OsIAA9* transgenic plant seedlings.** Four-day-old seedlings of Col wild type **(left)** and *OsIAA9* transgenic plants **(middle, right)** grown on vertically oriented plates were fixed with FAA overnight at 4°c, washed once with 50% ethanol, stained with Lugol’s solution for 1 min, and chloral hydrate solution for 2 min before photographs were taken immediately.

Starch granules in root tips play an important role during the sensing of gravity stimulus. Because the root gravitropic response was altered in the *OsIAA9* transgenic plants, we suspected that starch granule formation in the transgenic plants may be disrupted. To test if this is the case, we compared starch granule formation in wild type and the transgenic *Arabidopsis* seedlings by staining the roots with Lugol’s solution, which contains iodine that can react with starch granules to generate a visible bright blue color. We found that in wild-type root tips, strong staining was observed in several different layers of columella cells, while in root tips of the *OsIAA9* transgenic *Arabidopsis* seedlings, only slight staining was observed, and there is no clear stained cell layers could be observed (**Figure 7**).

## Discussion

Interplay of Aux/IAA proteins and ARFs regulates auxin signaling in *Arabidopsis* (Hagen and Guilfoyle, 2002). Characterization of gain-of-function mutants in *Arabidopsis* revolved that *Aux*/*IAA* genes regulated many aspects of plant growth and development (Nagpal et al., 2000; Rogg et al., 2001; Fukaki et al., 2002; Uehara et al., 2008; Ploense et al., 2009; Rinaldi et al., 2012). So far all experimental evidences suggest that Aux/IAA proteins in *Arabidopsis* and *rice* may regulate auxin signaling and plant growth and development in a similar manner (Jain et al., 2006; Nakamura et al., 2006; Jun et al., 2011; Zhu et al., 2011; Kitomi et al., 2012; Song and Xu, 2013). Our results in this report show that OsIAA9 is a canonical Aux/IAA protein, it regulates auxin signaling in a way similar to that of the canonical Aux/IAA proteins in *Arabidopsis*. However, unlike the *Arabidopsis* canonical Aux/IAA proteins have been studied so far, *OsIAA9* regulates lateral root formation and root gravitropic response when expressed in *Arabidopsis* in an unmutated form.

### OsIAA9 is a Canonical Aux/IAA Protein

All canonical Aux/IAA proteins contain four conserved domains. An LxLxL motif-containing domain, a degron-containing domain, and two domains required for protein–protein interaction (Ulmasov et al., 1997, 1999; Ramos et al., 2001; Tiwari et al., 2001, 2003, 2004; Dreher et al., 2006; Jain et al., 2006; Nanao et al., 2014).

Bioinformatics analysis showed that OsIAA9 is closely related to IAA31, a Domain II mutated type *Arabidopsis* Aux/IAA protein (**Figure 1B**). However, it contains all the features of a canonical Aux/IAA protein has, including the conserved KR residues crucial for 26 proteasome degradation of Aux/IAA proteins (Dreher et al., 2006), and the conserved W residue crucial for protein–protein interactions of OsIAAs and OsARFs (Ni et al., 2014) (**Figure 1B**). In consist with the canonical Aux/IAA protein features it has, OsIAA9 functions as a transcription repressor, its stability is affected by Auxin, it regulates auxin response repoter gene expression (**Figure 3**), and it interacted with ARF5 in transfected protoplasts (**Figure 4**). These results suggest that OsIAA9 is a canonical Aux/IAA protein, it regulates auxin signaling in a way similar to all other canonical Aux/IAA proteins.

### Expression of Unmutated *OsIAA9* in *Arabidopsis* Resulted in Auxin Related Phenotypes

So far all the *aux*/*iaa* mutants identified in *Arabidopsis* and rice with phenotypes are gain-of-function mutants with mutations occurred within Domain II of corresponding Aux/IAA proteins (Tian and Reed, 1999; Nagpal et al., 2000; Rogg et al., 2001; Fukaki et al., 2002; Tian et al., 2002; Knox et al., 2003; Uehara et al., 2008; Ploense et al., 2009; Jun et al., 2011; Zhu et al., 2011; Kitomi et al., 2012; Rinaldi et al., 2012). On the other hand, expression of mutated type, but not wild type *Arabidopsis* Aux/IAA genes in *Arabidopsis* resulted in auxin-related phenotypes (Park et al., 2002; Fukaki et al., 2005; Sato and Yamamoto, 2008). Expression of mutated or dominant mutation-type rice *Aux*/*IAA* genes in rice also resulted in auxin-related phenotypes (Nakamura et al., 2006; Song and Xu, 2013), suggest that stability of Aux/IAA proteins are crucial for their functions in regulating plant growth and development, and that function mechanisms of *Arabidopsis* and rice Aux/IAA proteins may be conserved.

Similar to other canonical Aux/IAA proteins, OsIAA9 functions as a transcription repressor, and its stability is affected by auxin as mentioned above. However, transgenic *Arabidopsis* plants expressing *OsIAA9* showed several auxin-related phenotypes, including increased lateral root formation and reduced root gravitropic response (**Figures 5** and **6**). Previously we have showed that expression of wild type *PtrIAA14.1*, a canonical Aux/IAA gene from poplar, resulted in phenotypes changes in *Arabidopsis* (Liu et al., 2015), but the phenotypes are different from that of *Arabidopsis* transgenic plants expressing *OsIAA9*. On the other hand, among all the five ARF activators, both PtrIAA14.1 and OsIAA9 interacted only with ARF5 in transfected protoplasts (Liu et al., 2015; **Figure 4**), indicating that interaction between ARF5 and OsIAA9 may contribute little, if any to the resulted phenotypes in the transgenic plants.

In summary, we showed that OsIAA9 is a canonical Aux/IAA protein, and it causes auxin-related phenotypes including lateral root formation and gravitropic response when expressed in *Arabidopsis*.

## Author Contributions

SW conceived the study. SL and SW designed the experiments. SL, QL, SL, NMP, and HT performed the experiments. SL and SW analyzed the data. SW drafted the manuscript. All authors read and approved the final manuscript.

## Conflict of Interest Statement

The authors declare that the research was conducted in the absence of any commercial or financial relationships that could be construed as a potential conflict of interest.

## Footnotes

1 http://phytozome.jgi.doe.gov/pz/portal.html

2 www.phylogeny.fr

3 http://rsbweb.nih.gov/ij

4 http://phytozome.jgi.doe.gov/pz/portal.html
